# Lineage divergence, local adaptation across a biogeographic break, and artificial transport, shape the genetic structure in the ascidian *Pyura chilensis*

**DOI:** 10.1038/srep44559

**Published:** 2017-03-16

**Authors:** Nicolás I. Segovia, Cristian Gallardo-Escárate, Elie Poulin, Pilar A. Haye

**Affiliations:** 1 Departamento de Biología Marina, Facultad de Ciencias del Mar, Universidad Católica del Norte, 1781421, Coquimbo, Chile; 2 Interdisciplinary Center for Aquaculture Research (INCAR), Departamento de Oceanografía, Universidad de Concepción, 4070386, Concepción, Chile; 3 Instituto de Ecología y Biodiversidad, Departamento de Ciencias Ecológicas, Facultad de Ciencias, Universidad de Chile, 7800003, Santiago, Chile

## Abstract

Marine benthic organisms inhabit a heterogeneous environment in which connectivity between populations occurs mainly through dispersive larval stages, while local selective pressures acting on early life history stages lead to non-random mortality, shaping adaptive genetic structure. In order to test the influence of local adaptation and neutral processes in a marine benthic species with low dispersal, in this study we used Genotyping by Sequencing technology to compare the neutral and putatively selected signals (neutral and outlier loci, respectively) in SNPs scattered throughout the genome in six local populations of the commercially exploited ascidian *Pyura chilensis* along the southeast Pacific coast (24°–42°S). This species is sessile as an adult, has a short-lived larval stage, and may also be dispersed by artificial transport as biofouling. We found that the main signal in neutral loci was a highly divergent lineage present at 39°S, and a subjacent signal that indicated a separation at 30°S (north/south), widely reported in the area. North/south separation was the main signal in outlier loci, and the linage divergence at 39°S was subjacent. We conclude that the geographic structure of the genetic diversity of outlier and neutral loci was established by different strengths of environmental, historical and anthropogenic factors.

Marine benthic species that inhabit patchy and heterogeneous substrates such as rock beds or sandy beaches are likely to be structured as metapopulations^1^,^2^. These species usually have dispersive larval stages that can act as propagules for connectivity between local populations. However, the amount of connectivity between their populations does not depend only on the presence and duration of dispersive larval stages^3^,^4^. Local adaptive pressures may differ drastically between local populations and may act as a settlement and survival sieve for early life stages of benthic marine organisms arriving to a local population^5^. Local adaptation can lead to strong differences between larval dispersal potential and realized dispersal^5^,^6^,^7^, by reducing recruitment and establishment of foreign individuals^5^,^8^,^9^ or by reducing the fitness of juveniles that establish and persist in the receiving population^10^.

The consequences of local adaptation in marine benthic species have been assessed with experimental methods such as reciprocal transplants and common garden experiments. These studies have shown there is differential survival between organisms of different origins in different environmental conditions^5^,^11^, and that there is non-random, genotype-dependent, selective mortality of recruits and post-colonization mortality^3^,^12^ that lead to high genetic differentiation among adult populations. In spite of the well-recognized effects of local adaptation on the geographic distribution of the genetic diversity of marine populations, there are few examples of phylogeographic studies that compare the spatial genetic signatures of natural selection and neutral evolutionary forces at a broad scale, allowing the evaluation of gene flow, genetic drift, natural selection and other mechanisms that may determine the geographic distribution of the genetic diversity of a species.

One experimental approach to analyze neutral and putatively adaptive signals is the use of genome scans to detect thousands of Single Nucleotide Polymorphism (SNP) loci scattered throughout the genome, some of which can be identified as having signals of natural selection by analyzing several individuals from different environments^13^. Spatial analyses of loci putatively affected by natural selection (i.e. outlier loci) provide insights into how adaptive variation could affect phylogeographic structure^14^,^15^ in contrast to the signal of loci that are neutral to natural selection. Studies focused on the assessment of phylogeographic signals of outlier and neutral loci in marine systems have been mostly developed for marine mammals and fishes^16^,^17^,^18^. These studies have generally shown that outlier loci possess a stronger signal of the genetic structure, as is expected by definition^19^,^20^. However, the main signal is mostly congruent between outlier and neutral loci. This kind of study in marine invertebrates is scarce; only recently a few studies have compared the phylogeographic signal of neutral and outlier loci using genome-wide procedures and have reported a mostly consistent structure between neutral and outlier loci in mussels^19^,^21^ and oysters^22^,^23^.

In this study we analyze neutral and adaptive spatial genetic structure of the benthic ascidian *Pyura chilensis* Molina 1972 along the Humboldt Current System (HCS) located in the southeast Pacific Ocean. The HCS is characterized by high productivity and regions with strong upwelling^24^. The wide geographic extent of the southeast Pacific coast and the cyclic variations of the HCS promote both temporal and spatial changes in population sizes and distributions of marine benthic species^24^. Despite being an almost linear coastline with apparent absence of geographic barriers between 18°S and 42°S, within the HCS coastal area there is a well-described marine biogeographic boundary located at 30–32°S. This biogeographic break is the limit of the distribution range of several marine species^24^, and phylogeographic studies of benthic marine taxa have shown that species with low dispersal capability have strong genetic discontinuity across the 30°S biogeographic boundary^25^,^26^. Life history attributes of marine benthic species and the oceanographic conditions seem to be the main factors that contribute to the geographic structure of the genetic diversity and differentiation in this geographic area^24^,^26^.

*Pyura chilensis* is a conspicuous inhabitant of the HCS widely distributed in the intertidal and shallow sub-tidal from 10°S to 44°S in the southeast Pacific coast^27^, and has been intensively exploited for human consumption^28^. Settlement occurs mainly on rough substrates and on the tunic of conspecifics^29^; it lives either as solitary individuals or as massive clumps of individuals (Supplementary Fig. S1). The species is hermaphroditic, with outcrossing as the main reproductive strategy^28^. *Pyura chilensis* has short-lived (12–24 hr) lecithotrophic tadpole-shaped larvae that provides a very low capacity to disperse between geographic areas along the HCS. In contrast to what has been reported for other sympatric benthic species of the HCS with low larval dispersal capacity^26^, *P. chilensis* did not show a genetic discontinuity at 30°S when analyzed with sequences of the mitochondrial gene Cytochrome Oxidase I (COI) and the nuclear gene Elongation Factor 1 alpha^30^. The lack of a genetic discontinuity at 30°S in the coast of Chile was novel for a benthic invertebrate with short-lived larvae. In fact, some species with planktonic larval durations of more than 2 days display significant genetic divergence across the 30°S biogeographic boundary^26^. By uncovering abundant neutral and adaptive genetic variation in *P. chilensis* it may be possible to detect variation at 30°S, as would be expected in a benthic species with a low dispersal potential.

An alternative means of dispersal for ascidians such as *P. chilensis* is transport in artificial structures such as ship and boat hulls, where the species can grow attached^31^,^32^,^33^. Although this has not been specifically assessed for *P. chilensis*, the species has often been seen on the hulls of boats of artisanal fisheries, on buoys, ropes and on any other marine facility that provides substratum as an important part of the fouling community^34^,^35^. This has been hypothesized as one of the mechanisms behind the low degree of genetic differentiation of the COI gene along a broad geographic area and across the 30°S biogeographic break^30^; similarly, anthropogenic transport has often been suggested as a main dispersal and invasion mechanism for other ascidians^30^,^31^,^32^,^33^.

Another relevant aspect about *P. chilensis* is the genetic structure of the species based on COI sequences. The COI phylogeographic structure of the species was characterized by three mitochondrial haplogroups, two of which were sympatric and widespread, and a third that was highly divergent from the other two and geographically restricted, predominantly found between 39°S and 42°S, being most abundant at 39°S in Los Molinos (LM)^30^. Based on COI sequences, LM was the most differentiated population because it harbored a high proportion of individuals of a unique and divergent haplogroup. Here we evaluate the divergence of the LM population of *P. chilensis* using neutral and putatively adaptive SNP loci, and explore if natural selection follows the same signal as neutral sequence divergence, i.e. if LM is also highly differentiated with putatively adaptive loci. Even though physical transport of a few individuals per generation between local populations should be enough to maintain genetic homogeneity in the whole genome at both individual and population levels^3^,^36^, non-neutral genetic variation affects fitness, influencing recruitment and survival of individuals in the variable marine environment. Thus, lack of spatial genetic differentiation at neutral or nearly neutral loci does not imply that outlier loci will provide the same signal^36^.

Here we evaluate the spatial genetic structure of neutral and outlier SNP loci in six populations of *P. chilensis* covering from 24°S to 42°S, and across the 30°S biogeographic discontinuity in the southeast Pacific. We aim to assess the influence of contrasting evolutionary forces, including natural selection (local adaptation), isolation and drift (lineage divergence at 39°S), and gene flow (artificial transport), on the spatial genetic structure of the benthic ascidian *P. chilensis* in the HCS.

## Results

### SNP discovery and outlier detection

Extracted DNA was of high quality, with average 260/280 and 260/230 ratios of 1.96 ± 0.01 and 2.15 ± 0.03, respectively. Genotyping by Sequencing (GBS) of 90 individuals of *P. chilensis* (15 of each of six local populations; Table 1) gave a total of 202,494,184 reads. Of these, 169,855,853 produced good quality barcode reads. The FastQC results showed a mean per-base quality score (*Phred* score) of 36 and a mean quality score of 33.12. After applying the trimming and demultiplexing approach, 163,692 tags were produced with a mean of 4.38 reads per individual. The UNEAK pipeline detected a total of 81,837 SNP. After filtering with a minor allele frequency (MAF) of 4.4% and a minimum call rate (mnC) of 90%, the resulting dataset consisted of 2,332 SNPs.

Using BAYESCAN with a False Discovery Rate (FDR) correction of *q-values* of 0.05, 81 of the 2,332 loci uncovered showed F_*ST*_ values greater than expected under a neutral distribution (*q-values* < 0.05) (Supplementary Fig. S2). These 81 loci were analyzed separately; thus, there were two datasets, one with the 81 outlier SNP loci and one with 2,251 neutral SNP loci.

### Population genetic diversity and population structure analyses

Mean expected and observed heterozygosity (H_E_ and H_O,_ respectively) were mostly similar between neutral and outlier loci. H_E_ mean values ranged from 0.290 to 0.294 and 0.250 to 0.358 for neutral and outlier loci, respectively. Mean H_O_ values ranged from 0.169 to 0.304 and 0.143 to 0.345 for neutral and outlier loci, respectively (Table 2). The mean allelic richness (A_r_) within population ranged from 1.560 to 1.662 for neutral loci and 1.511 to 1.626 for outlier loci. Northern populations generally showed lower values of A_r_ in neutral loci and similar values between all sampled populations in outlier loci; LH and LM were the sites with the lowest values. The exact test for HWE deviations showed that after FDR correction, only the LM site showed significant deviation (Table 2).

Most of the population-pairwise F_*ST*_ values for both neutral and outlier loci were significant, with the exception of PA and CP in neutral loci (Table 3), indicating that *P. chilensis* harbors neutral and adaptive population differentiation. F_*ST*_ values ranged between 0.003–0.417 and 0.027–0.692 for neutral and outlier loci, respectively (Table 3). For neutral loci F_*ST*_ values were generally low (0.003 to 0.049) with the exception of Los Molinos (LM) that was highly differentiated with values ranging between 0.393 and 0.417, which are an order of magnitude greater than all the rest of the values. In contrast, all F_*ST*_ values calculated with outlier loci were, as expected, relatively high; remarkably, in neutral loci LM did not show higher levels of differentiation than the other population pairwise comparisons. In the outlier loci population-pairwise differentiation matrix, the values between pairs from the northern (PA, 26°08′S; CP, 27°44′S; LH, 29°58′S) *versus* southern (TH, 36°38′S; LM, 39°50′S; AC, 41°52′S), *i.e*. north/south of 30°S, showed the highest differentiation values (Table 3).

Using AMOVA analysis the separation of populations north and south of 30°S explained 2.91% and 48.73% of the variation for neutral and outlier loci, being significant only for outlier loci. The analysis of the separation of LM from the rest of the populations explained 39.5% and 8.74% of the genetic variation for neutral and outlier loci, respectively, and was only significant only for neutral loci (Table 4).

**Figure 1:**
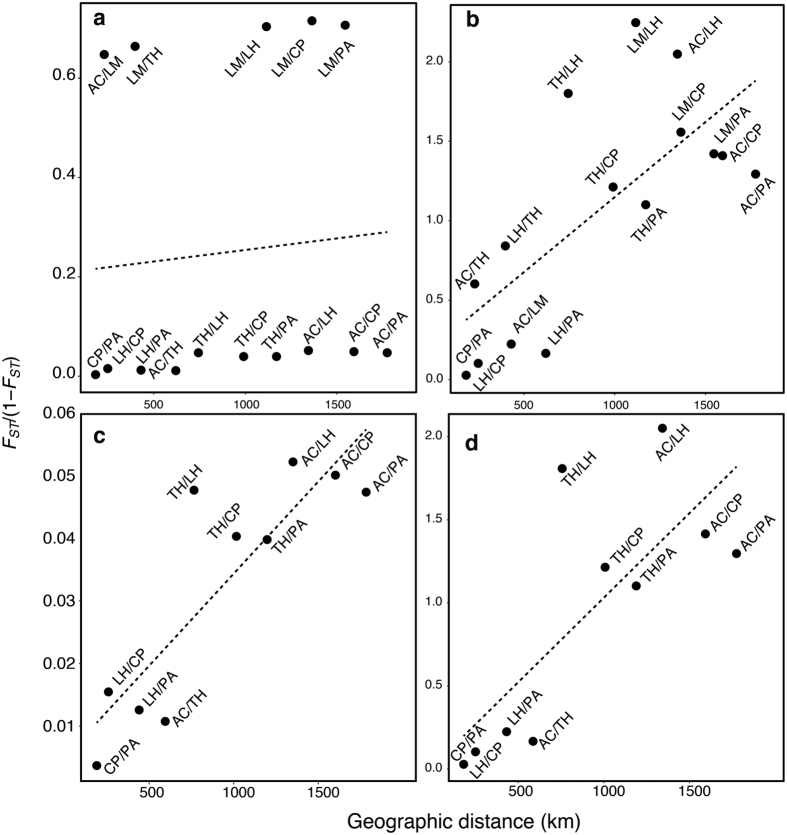
Relationship between genetic differentiation and geographic distance for neutral and outlier loci of *Pyura chilensis*. Relationship between linearized genetic differentiation (F_*ST*_) and geographic distance (Km) along the Humboldt Current System. (**a**) Relationship using neutral markers, (**b**) for outlier markers and (**c**) and (**d**) show the relationship of neutral and outlier makers, respectively, excluding LM from the analyses. All the pairwise comparisons are marked in the graph.

Mantel tests did not detect a significant relationship between the linearized genetic distance and geographic distance for either neutral or outlier markers, with nearly significant *p-values* (r = 0.034, *P *=* *0.089 and r = 0.75, *P *=* *0.068, respectively) (Fig. 1a,b). Excluding LM from the analysis of neutral loci indicated a marginally significant relationship between genetic and geographic distance (r = 0.85, *P* = 0.048) (Fig. 1c). In the case of outlier loci, this led to virtually the same result as when LM was included in the analysis (r = 0.81, *P = *0.064) (Fig. 1d).

**Figure 2:**
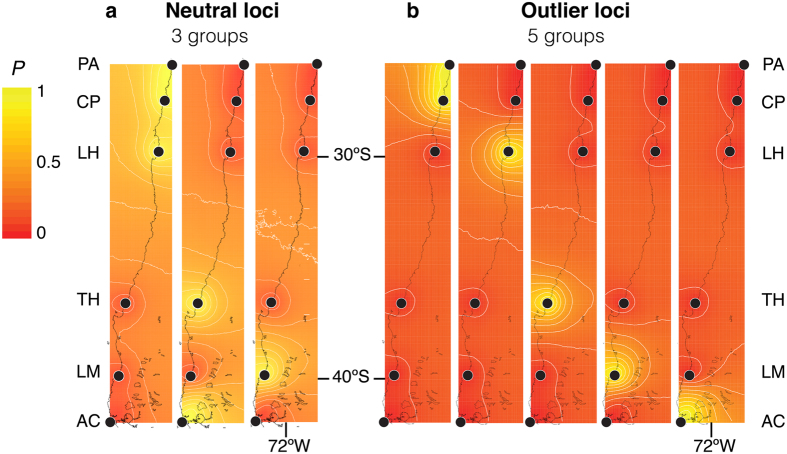
Spatial genetic structure of *Pyura chilensis* based on GENELAND analyses. Each map indicates the posterior probability of belonging to one of the three groups found in neutral loci, or the five groups found in outlier loci. Black dots represent the coordinates of the sample sites, and the x and y-axes correspond to geographic coordinates.

In order to detect spatial population genetic structure we used the GENELAND software, which revealed the presence of three groups in the neutral loci dataset. One corresponded to the three northern localities PA, CP and LH; the second included TH and AC and the third was LM (Fig. 2a). Five groups were detected using outlier loci; one group contained PA and CP, and each other local population was assigned to a single genetic group (Fig. 2b).

**Figure 3:**
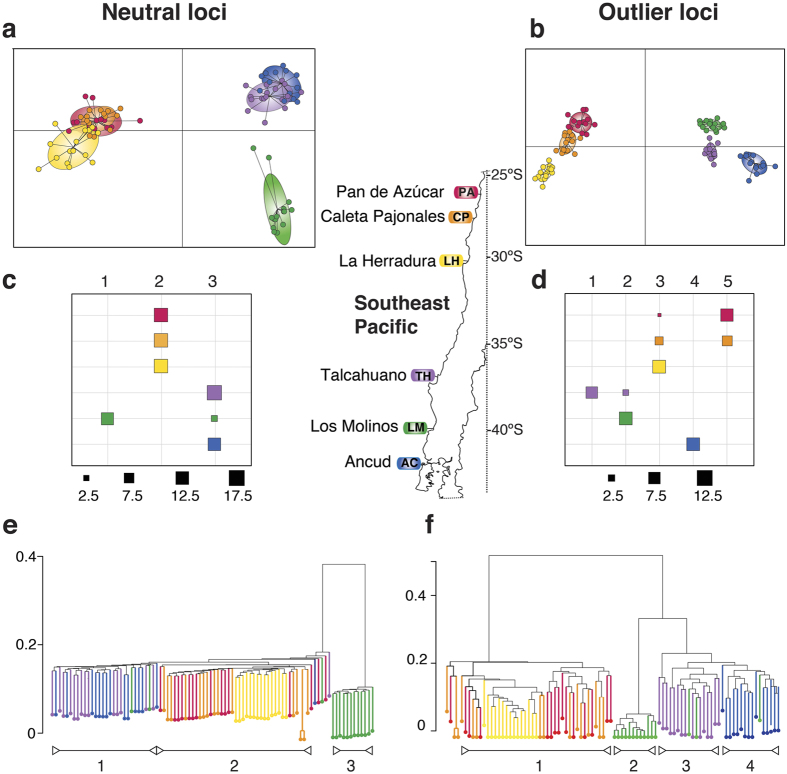
Genetic Clustering of neutral and outlier SNP loci for *Pyura chilensis.* (**a,b**) Discriminant Analyses of Principal Components (DAPC) scatterplot showing the first 2 principal components for *K* = 3 and *K* = 5 (neutral and outliers, respectively). Colors represent the sampling location of each individual. (**c,d**) Proportion of individuals that belong to each cluster based on the DAPC analysis using a BIC criterion with geographic information, (**e**,**f**) Identity-by–state (IBS) pairwise relatedness dendrogram for all possible pairs of individuals. The dendrogram shows the three clusters found for neutral markers and the four clusters for outlier markers.

Cluster analyses confirmed the disparate signals of neutral and outlier loci. In the clustering approach of the neutral loci analysis using DAPC (considering the sampling site as a prior), the first principal component axis (vertical) separated LM from all the rest of the populations (Fig. 3a). The second axis (horizontal) separated the north/south of 30°S, with an overlap in the three populations of the north (PA, CP, LH) and an overlap of TH and AC in the south (LM did not show overlap) (Fig. 3a). Thus, the main signal derived from the DAPC analysis of neutral loci was the separation of LM from the rest of the populations and there was a weaker signal associated with the 30°S biogeographic boundary. In outlier loci, north/south of 30°S were well separated in the horizontal axis, with an overlap of PA and CP in the north of TH and LM in the south. The vertical axis separated the PA/CP group from LH, and LM from AC and TH (Fig. 3b).

As with GENELAND analyses, DAPC performed without considering the information of the geographic origin of the samples, and based on the BIC, detected three and five groups as the optimal separation for neutral and outlier loci, respectively (Fig. 3c,d). For neutral loci, the three northern populations appeared in the same cluster, and in the south, TH and AC appeared in the same cluster but LM was clearly separated, with the exception of three individuals that clustered with TH and AC (Fig. 3c). In outlier loci the five groups were mostly consistent with the geographic origin with the exception of PA/CP, which formed a single cluster (Fig. 3d). The third group was composed mainly of individuals of LH (Fig. 3d).

Dendrograms constructed with Identity-by-Site (IBS) without incorporating geographic information detected three and four clusters for neutral and outlier loci, respectively, providing a similar pattern of differentiation as previous analyses (GENELAND, DAPC). With neutral loci LM appeared as a distant separate cluster, while the two other clusters were closer together. These are the three localities north of 30°S and the southern localities TH and AC (Fig. 3e). Again, three individuals of LM appeared together with the individuals sampled in TH and AC. For outlier loci, the north/south of 30°S were well separated (Fig. 3f). The analysis detected two groups in the northern cluster. The first group was composed mostly of individuals of LH and of individuals of PA and CP in lower frequency; the second group was composed exclusively of individuals of PA and CP (Fig. 3f). In the southern cluster, most of the individuals from LM (except three) conformed a well-separated cluster and the rest of the southern individuals including those three individuals from LM formed a single cluster (Fig. 3f).

**Figure 4:**
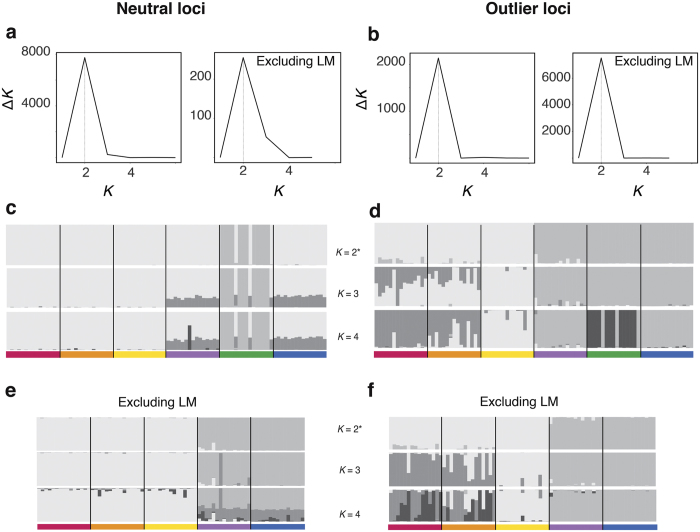
Bayesian clustering inferred with STRUCTURE for *Pyura chilensis.* (**a**,**b**) Delta *K* results for neutral and outlier loci including and excluding Los Molinos (LM) to infer optimal separations according to Evanno *et al*.^67^, (**c**–**f**) Bayesian assignment probabilities of individuals of inferred with STRUCTURE with the entire dataset and excluding LM from the analyses. Each column represents one individual, with the color representing the membership proportion of each of the clusters. The vertical line represents the delimitation of each sampled local population. Graphs show the results for *K* = 2–4. The optimum *K* was denoted by an asterisk.

With Evanno’s method, *K* = 2 was determined as the optimal separation for both data sets in analyses based on STRUCTURE (Fig. 4a,b). For neutral loci one cluster included most of the individuals of LM (12 of 15 individuals), while the second included all other sampling sites plus the three remaining individuals of LM (Fig. 4c). A slight north/south of 30°S separation appeared as a subjacent signal when forcing *K* = 3 and *K* = 4 (Fig. 4c).

The optimal clustering separation for outlier loci, *K* = 2, corresponded to a separation between north/south of 30°S (Fig. 4d). These results indicate that the optimal *K* of neutral and outlier loci provide a different spatial genetic structure of *P. chilensis*. Forcing *K* = 3 for outlier loci divided the northern cluster in two, one group with PA and CP with strong influence of a third group located mainly in LH. Only when forcing *K* = 4 an additional cluster appeared in the south corresponding to 12 of the 15 individuals of LM (Fig. 4d). The LM cluster was evident with optimum *K* for neutral loci while it appeared with non-optimum *K* = 3–4 for outlier loci. Exploring from *K* = 5 to *K* = 7, there was no significant difference in the general pattern compared to *K* = 4 in both datasets (results not shown). When LM was excluded from the analyses, the optimal separation also corresponded to *K *=* *2 (Fig. 4a,b). For both data sets, the clusters formed corresponded to the north/south of 30°S separation (Fig. 4e,f).

## Discussion

The use of putative selected markers (outlier loci) in marine benthic invertebrates has usually increased the strength of detected phylogeographic structure with respect to neutral loci, as is expected, albeit the general patterns remain the same^19^,^21^,^22^,^23^. In contrast to this generalization, in the present study neutral and outlier loci showed contrasting spatial genetic structure patterns in the ascidian *Pyura chilensis* along 18° degrees of latitude in the Southeast Pacific coast. Neutral loci had a main signal associated with the lineage history and genetic differentiation of Los Molinos (LM) (39°S), and had a subjacent weaker signal of genetic structure across the 30°S biogeographic boundary. Inversely, outlier loci showed a stronger signal across 30°S and a weaker subjacent signal of lineage divergence at 39°S (LM). The two sets of loci showed the same two phylogeographic patterns, structure across 30°S and 39°S, but their strength was in reverse order. Additionally, the degree of neutral genetic differentiation detected was lower than expected from the short larval duration (2 days) and did not adjust to a pattern of isolation by distance (IBD), suggesting that artificial transport also contributes to the neutral genetic structure of *P. chilensis* along the Humboldt Current System (HCS). SNP sampling and detection of putatively adaptive loci allowed the evaluation of the contributions of several evolutionary mechanisms in the phylogeographic structure of *P. chilensis.* We detected a strong adaptive signal of differentiation at the 30°S biogeographic break, a strong neutral signal associated with the divergent lineage present at 39°S, and the influence of artificial anthropogenic transport.

LM had pairwise genetic differentiation values with neutral loci one order of magnitude higher than the rest. This is explained by 12 of the 15 individuals from LM that were highly differentiated from all other individuals of all other sites, independent of whether the analytical approximation used was frequency or distance-based. These differentiated individuals belonged to the divergent mitochondrial DNA haplogroup previously identified in LM^30^ (Supplementary Table S1). The extent of the geographic distribution of the divergent lineage that is present in LM needs to be further investigated with an intensive sampling between TH and LM and in the vicinity of LM towards the southern AC locality. It is likely that migration to LM from well-differentiated populations may account for the detected genetic differentiation. The apparent absence of admixture between individuals of LM belonging to the two different clusters as shown by STRUCTURE (Fig. 4e) suggests that these differentiated individuals found in LM may correspond to a reproductively isolated unit. Although these results clearly identified different genetic groups, the existence of a cryptic species in this area should be further investigated, including molecular and morphological systematic studies^37^, before drawing taxonomic conclusions.

As expected, outlier loci showed higher levels of differentiation between all local populations than neutral loci, and the genetic signal provided by outlier loci differed from the neutral signal. The main signal in outlier loci was a strong differentiation north/south of the 30°S biogeographic break, explaining almost half of the total variation in the data. In contrast to the signal detected with neutral markers, outlier loci did not show a main signal associated with the genetic differentiation of LM. Still, the main signal of differentiation north/south of 30°S in outlier loci did not completely expunge the influence of the differentiation of LM; LM appeared as a distinct group when forcing a third suboptimum clustering (*K* = 4) in STRUCTURE (Fig. 4f), and within the southern cluster the IBS dendrograms showed that the same 12 individuals from LM were clearly separated from the other individuals from south of 30°S (Fig. 4f). Additionally, Hardy-Weinberg Equilibrium (HWE) was prevalent; LM was the only population with significant deviations. We explored HWE excluding the 3 individuals of LM that are not highly divergent from the rest of the data set, and after the FDR correction, LM did not deviate from HWE, suggesting that lack of HWE in LM was likely due to a Wahlund effect, in agreement with presence of two divergent lineages in the site^30^. The fact that the 12 divergent individuals from LM did not appear as a separate and external cluster for outlier loci may reflect that natural selection acts globally on these taxa, responding more to environmental constraints than lineage evolution. In other words, the coherence between geographic localities and genetic distribution in outlier loci suggests that selection, independent of the evolutionary history of the lineage, maintains the genetic cohesion of the species mainly based on environmental constraints rather than on lineage history. As genetic variation in neutral markers may reflect historical and contemporary connectivity among populations^38^, selected markers may reflect adaptive processes associated with differences in environment conditions experienced by the species along the HCS.

IBD including the all data set was nearly significant and it was marginally significant when LM was excluded from the analysis. However, STRUCTURE analyses showed that when LM was excluded, most of the genetic variation was explained by the differentiation to the north and south of 30°S, with optimum *K* = 2. Therefore, Mantel test results may just reflect a main genetic discontinuity between north/south of 30°S instead of a progressive decrease in migration rate among neighboring sites relative to geographic distance.

Phylogeographic patterns of marine invertebrates along the HCS have shown that the biogeographic boundary at 30°S is a historical discontinuity and that species with low dispersal potential retain the genetic signal and have a concordant phylogeographic break^26^. However, this structure had not been previously detected in *P. chilensis* using sequence data^30^. This is not surprising if we consider that neutral loci revealed that the structure at 30°S is subjacent to a stronger signal of lineage divergence at 39°S, which was the same signal detected with sequence data^30^. The clear differentiation detected by outlier loci between north/south of 30°S is likely linked to the historical and contemporary existence of a marine biogeographic boundary^24^. The contemporary influence of this discontinuity is likely attributed to the heterogeneous environmental conditions that differ markedly north and south of 30°S, mainly, the kinetic energy of the ocean^39^, differences and seasonality in upwelling-favorable winds^40^, and differences in the influence of freshwater on coastal waters^24^. The oceanographic differences between north/south of 30°S could imply that in sessile organisms, such as *P. chilensis,* the effective connectivity (recruitment and survival of juveniles) is restricted, despite the genetic evidence that there is transport of individuals between the two areas^30^. Considering that neutral phylogeographic structure of benthic marine species across 30°S is strongly associated with life history^26^, *P. chilensis* showed phylogeographic structure across 30°S as expected, although it had less differentiation across 30°S than other species with similar dispersal potential based on larval duration.

Differentiation was greater than had been previously reported for *P. chilensis*^30^,^41^, and also greater than what has been reported for other ascidians that have intensive connectivity driven by anthropogenic transport^31^,^32^,^33^,^42^. The influence of anthropogenic transport was evident from the low, albeit significant differentiation detected between distant local populations, such as the southernmost site analyzed AC and all the northern sites, and general the lack of IBD. The single exception to the significant genetic differentiation detected with neutral loci was the population pairwise genetic differentiation between PA and CP, that was low and non-significant. A possible explanation is that PA and CP are separated by less geographic distance than any other pair of populations, permitting higher gene flow between them than with the rest of the sites. However, short larval life history alone is unlikely to maintain high connectivity between PA and CP, suggesting that artificial transport may be enhancing connectivity between these sites, in agreement, shipping trajectories in the area between PA and CP seem to be geographically continuous and intense (Supplementary Fig. S3).

Physical transport as biofouling may have allowed the latitudinal expansion of *P. chilensis* after periods of isolation during the Pleistocene^30^, but the invasive potential is likely less than in other invasive ascidians. In part this may be explained by the susceptibility of the species to abiotic disturbances^34^, the low and strongly seasonal recruitment rate^43^, and, compared to colonial ascidian growth forms (e.g. *Diplosoma* sp.), a slower growth rate^34^. Higher growth and recruitment rate and rapid reproduction are important life history features in invasive ascidians that allow larvae to colonize open surfaces quickly^44^. Valdivia *et al*.^34^ suggested that *P. chilensis* is a good example of a strong competitor with relatively low colonizing ability.

Shipping routes (Supplementary Fig. S3) may also explain the greater differentiation of LM and of LH. LM is the most differentiated population, with a divergent group of individuals, and fittingly, there is very low maritime traffic in the area around LM. There is also low traffic inside La Herradura Bay (LH). The lower differentiation of LH than LM may be explained by the distance from the open coast, which is much smaller in LH, probably allowing slightly greater connectivity by maritime routes. Additionally, differentiation of LH was enhanced by the high proportion of individuals belonging to a distinct mitochondrial haplogroup^30^ (Supplementary Table S1). Other sites had from 0 to 4 individuals of this haplogroup, while LH had 7 of the 15. The relatively high genetic differentiation of LH is likely shaped by the lower maritime traffic present in the area, enhancing genetic differentiation.

Although the results are robust and allow inferring the spatial genetic structure of loci affected by contrasting evolutionary forces, it is important to consider alternative explanations associated with the quality of information provided by the data. For example, identified outlier SNP loci may be linked to loci that are the direct target of natural selection instead of natural selection directly operating on them^45^. Additionally, spatial autocorrelation can cause random associations between the environment and the genetic structure of a species as a result of dispersal following IBD or demographic processes^46^. Some coding regions may have greater genetic-environmental associations than others due to deleterious mutations being selected against in all the study area (purifying selection), and not because advantageous mutations are being selected for in a particular environment (local adaptation^47^). Other factors that may cause loci to behave as outliers are recombination, sampling design, and locus-specific effects^46^,^48^.

Demonstrating that local adaptation has occurred involves not only the detection of variation at outlier loci, but also the understanding of the functional differences between alleles of individual candidate genes^48^. The knowledge of the physical linkage of SNPs allows the identification of genomic regions with unequal response to natural selection^45^. Since *P. chilensis* is a non-model organism, information on candidate genes is not currently available. In spite of the possible caveats, 3.5% (81) of the total retained SNPs (2,332) showed low and significant *q-values* and higher levels of F_*ST*_ for 90 individuals, allowing us to distinguish individuals genetically between sampling locations at a relatively small geographic scale, and suggesting that significant differences could be due to positive selective pressures^49^ that differ north/south of 30°S. The percentage of outlier loci detected is proportionally consistent with other studies that show high variation between local populations^19^,^22^,^23^,^50^,^51^. Further studies are needed to evaluate the genes involved in the putatively adaptive genetic structure detected here, and determine if putatively selected loci are actually under positive selection due to different environmental conditions north and south of the biogeographic boundary at 30°S in the southeast Pacific. Here we showed that even in the presence of a highly divergent lineage, the putatively selected signal could recover a pattern of genetic structure that masks the evolutionary history of the species.

In this study, we obtained several SNPs scattered throughout the genome and analyzed and compared the genetic structure of neutral and putatively adaptive outlier loci in *Pyura chilensis*. The comparison allowed us to conclude that both outlier and neutral loci were diverse and variable in space, and that both have shaped the genetic diversity of *P. chilensis*. The geographic structure of the genetic diversity of outlier and neutral loci was established by different strengths of environmental, historical and anthropogenic factors.

## Methods

### Sample collection and DNA extraction

Samples of *Pyura chilensis* were obtained by local fisherman from six sites along the Humboldt Current System in the Southeast Pacific Ocean (Table 1). Fisherman sampled individuals from natural substrates in areas with abundant *P. chilensis*. In all sites samples were taken in the shallow subtidal. Even though the degree of relatedness of individuals in a clump has not been assessed, in order to avoid possible effects of relatedness several clumps were obtained, all separated by at least 2 meters. Once in the fishing port, we randomly chose 40–45 clumps or isolated individuals in order to obtain one sample from each, and of those 40–45, we randomly picked 15 per site for this study. Mantle tissue (0.2 g) from each individual was used to extract DNA using the DNeasy Blood^®^ & Tissue Kit (QIAGEN^®^, USA) according to the manufacturer’s instructions. Quantity/purity of DNA was measured in Nanodrop 2,000 (Thermo, USA).

### Genotyping by Sequencing and SNP discovery

Samples were sequenced in the Biotechnology Center of the University of Wisconsin, USA using Genotyping by Sequencing (GBS)^52^, a method widely used in non-model species and that has proven to be a useful tool to distinguish between local populations at the genome level in marine benthic invertebrates^20^. This is a Reduced Representation Sequencing (RRS) method, which is based on the use of restriction enzymes that reduce the complexity of the genome, with the additional advantage that it can be performed without prior knowledge or reference genomes. This technique allows sequencing a large number of short genomic regions in several individuals^45^, permitting the genotyping of a large number of SNPs randomly located throughout the genome. Previous to sequencing, two additional enzymes were tested (*PstI, PstI/TaqI*) and finally *ApeKI*^53^ was chosen due to the wide genome distribution of flanking regions described for other species and avoiding repetitive zones^53^.

Libraries were prepared for the DNA of 15 individuals of each population. Following the restriction enzyme digestion, DNA fragments were ligated to a unique barcode adaptor for each individual. The library was prepared in a 96-well plate with 6 wells as blank, using a Hiseq2000 (Illumina, USA) platform, which sequences reads of approximately 100 base pairs (bp). The reads were visualized in FastQC version 0.10.1^54^ for quality checks.

All the data were prepared and analyzed using the pipeline Universal Network-Enabled Analysis Kit (UNEAK^55^) using TASSEL v.3^56^ that is specially designed for species with no reference genome. TASSEL-UNEAK is a network based SNP detection algorithm that may be less flexible than other pipelines in aspects like read trimming and parameters for *de novo* locus identification^57^, reducing the potential number of total SNPs detected in the dataset. However, TASSEL-UNEAK has proven to be a useful, reliable and reproducible tool for demultiplexing and processing sequence data obtained through GBS for non-model species^55^. With the UNEAK pipeline, the dataset was demultiplexed and the reads were trimmed to 64 bp to remove the barcode sequence and the error-prone tail of the sequences. After the network filtering, identical reads were aligned as tags using an error tolerance rate of 0.03 in order to minimize the chance that real tags were discarded as sequencing errors and to remove potential paralogs before the SNP calling. This filtering is a goodness-of-fit χ^2^ (α = 0.05) based on the null hypothesis that the counts of two paired tags of a SNP are equal in all heterozygous individuals^55^.

Additionally, a minor allele frequency (MAF) of 0.044 was used to filter the number of SNP loci whose variability was well represented in the 90 analyzed individuals. This value was determined empirically based on the probability that at least 8 of the 180 possible allele calls (4.4% of the alleles detected in 90 individuals) correspond to the minor allele. Values of minimum call rate (mnC) from 75 to 90% were tested previous to the final SNP calling, finally choosing the most exigent value of mnC (90%) in order to remove all SNPs containing 90% or more “N” values, which designate those individuals for which no allele was assigned for the locus or that were covered by no more than one tag and because of the amount of missing data detected, preserving a reduced but robust set of SNPs with reliable genotypes without excluding individuals from the analysis. These restrictive filters were performed because all loci were sampled at a mean coverage of ≥10x per individual. Under this condition, in theory each individual had in average 10 or more copies of a locus.

### Outlier detection

To identify the SNPs putatively under diversifying selection we used the F_*ST*_ outlier approach, which is based on the estimation of those loci with greater values of F_*ST*_ (outliers) with respect to the expected values under a neutral distribution. We used the software BAYESCAN 2.1^16^ for F_*ST*_ outlier detection, because it has been reported to have lower rates of false positives with respect to other similar software^58^. BAYESCAN uses a Bayesian framework to calculate the posterior probability that any given locus is under selection^16^. A total of ten separate runs were performed, from 50,000 to 500,000 iterations with a 10% burn-in period to assure the convergence of the MCMC. After the runs, an FDR correction of *q-values* of 0.05 was applied in BAYESCAN to avoid the occurrence of false positives. The data set was then separated into two parts, one with the outlier SNPs, which are loci putatively under diversifying selection^16^, and one with neutral SNPs, which are all the rest of the loci that have F_*ST*_ values that match the expected under a neutral distribution.

### Population genetic diversity analysis and genetic structure

From here on, all analyses were performed separately for each of the data sets (neutral and outlier loci).

To evaluate the differences in genetic diversity across the study area in each data set, we calculated the expected heterozygosity, observed heterozygosity and allelic richness with rarefied allele counts, using the HIERFSTAT package version 0.04–22^59^ in R v 3.22^60^. Exact tests for Hardy-Weinberg Equilibrium deviations for outlier loci were calculated using GENEPOP version 4.6^61^. Multiple comparisons of HWE were corrected using a FDR.

Pairwise F_*ST*_ and their significance were determined using a permutation test (10,000) in ARLEQUIN Version 3.5^62^. ARLEQUIN was also used to perform an Analysis of Molecular Variance (AMOVA) using the north/south of 30°S biogeographic boundary, and the separation of LM as two *a priori* groupings. Additionally, a Mantel test with 10,000 permutations was carried in out in ARLEQUIN 3.5 to test if there is a pattern of isolation by distance, using the relationship between the geographic distance (Km) and Slatkin’s linearized genetic distance (F_*ST*_).

Spatial population genetic structure was assessed using the Bayesian clustering algorithm implemented in GENELAND v 3.1.4^63^. This analysis uses spatial coordinates to separate allele frequencies that can be detected with departure from Hardy-Weinberg and linkage equilibrium into *K* clusters. An independent model of allele frequencies was carried out for both data sets, with 10 independent runs with 1e^6^ iterations and a 10% burn-in period, varying *K* from 2 to 6. The optimum *K* was inferred from the posterior individual membership probability of each genetic group. Posterior probabilities of membership were plotted with the *shapefiles* of the coastline available in the database of GEOdas (NOAA), filtering in the study area using GEOdas Coastline Extractor v 1.1.3.1 (https://www.ngdc.noaa.gov/mgg/geodas/geodas.html).

A clustering approach was used with a discriminant analysis of principal components (DAPC) in the package *adagene*^64^ in R v 3.22^60^. In DAPC, a principal components analysis of the multilocus genotypes of the individuals was calculated and then a discriminant analysis was carried out using the PC scores. These analyses are based on the detection of the number of clusters that minimizes between-group variation^64^ using the k-means and a Bayesian Information Criterion (BIC) to identify the optimal number of clusters in the data, not assuming underlying structure in population subgroups or panmixia as other similar approaches do. The number of clusters in each data set was determined using 10e^7^ iterations. To avoid unstable assignments in each cluster, 30 PCs were retained (N_total_/3), using all the 6 discriminant functions. We determined the optimal number of PCs using 1,000 simulations to execute the final DAPC.

To infer SNP-based relatedness structure within populations, an analysis based on pairwise IBS (identity-by-state) was used. This approach uses the information of the genotypes to calculate the probability that two alleles in the same segment have the same ancestor. A cluster analysis of the matrix generated with the IBS pairwise coefficients between all possible pairs of individuals was performed using a permutation score of 10,000 to determine how many groups were present in each data set. This was performed in the package SNPRelate^65^ in R environment v 3.22 (R Core Development Team 2015).

To determine the number of genetic groups we used STRUCTURE 2.3.4^66^. Clusters (*K*) varied from one to seven, corresponding to the number of analyzed locations plus one. Ten replicate runs, each with 500,000 MCMC with a 10% burn-in period, were performed under the admixture and correlated frequency model. The most probable *K* value was inferred based on the delta *K* method proposed by Evanno *et al*.^67^.

### Data Accessibility

SNP calls for all data set (2,332 SNPs) are available in a VCF file that has been deposited in DataDryad (doi:10.5061/dryad.0s87v).

## Additional Information

**How to cite this article:** Segovia, N. I. *et al*. Lineage divergence, local adaptation across a biogeographic break, and artificial transport, shape the genetic structure in the ascidian *Pyura chilensis. Sci. Rep.* **7**, 44559; doi: 10.1038/srep44559 (2017).

**Publisher's note:** Springer Nature remains neutral with regard to jurisdictional claims in published maps and institutional affiliations.

## Supplementary Material

Supplementary Information

## Figures and Tables

**Table 1:** Sampled local populations of *Pyura chilensis*.

Location	Coordinates	Code	N
Pan de Azúcar	26°08′S; 70°39′W	PA	15
Caleta Pajonales	27°44′S; 71°02′W	CP	15
La Herradura	29°58′S; 71°21′W	LH	15
Talcahuano	36°38′S; 71°21′W	TH	15
Los Molinos	39°50′S; 73°23′W	LM	15
Ancud	41°52′S; 73°50′W	AC	15

Sampling localities, coordinates, code, number of successfully analyzed individuals for genome scans for *Pyura chilensis* in the Southeast Pacific coast.

**Table 2:** Population genetic statistics.

Site	Proportion of polymorphic loci		*A*_*r*_		H_O_		H_E_		HWE (Outlier)		
	Neutral	Outlier	Neutral	Outlier	Neutral	Outlier	Neutral	Outlier	χ^2^	df	*P-value*
PA	0.2	0.23	1.560 ± 0.43	1.639 ± 0.46	0.271 ± 0.21	0.306 ± 0.20	0.294 ± 0.16	0.338 ± 0.17	89.8	96	0.66
CP	0.59	0.59	1.579 ± 0.42	1.619 ± 0.46	0.304 ± 0.23	0.318 ± 0.20	0.295 ± 0.16	0.358 ± 0.16	118.17	90	0.02
LH	0.48	0.65	1.581 ± 0.42	1.511 ± 0.47	0.305 ± 0.23	0.319 ± 0.26	0.291 ± 0.16	0.299 ± 0.18	89.46	74	0.11
TH	0.37	0.4	1.605 ± 0.40	1.666 ± 0.45	0.285 ± 0.22	0.334 ± 0.19	0.290 ± 0.16	0.336 ± 0.15	69.14	102	0.99
LM	0.46	0.44	1.662 ± 0.39	1.554 ± 0.46	0.169 ± 0.18	0.143 ± 0.15	0.288 ± 0.15	0.250 ± 0.14	197.41	76	**<0.001**
AC	0.57	0.61	1.604 ± 0.41	1.626 ± 0.46	0.298 ± 0.23	0.345 ± 0.21	0.289 ± 0.16	0.340 ± 0.17	57.08	92	0.998

Proportion of polymorphic loci and allelic richness (A_r_) for neutral/outlier loci, observed (H_O_) and expected (H_E_) heterozygosity and deviations of Hardy-Weinberg Equilibrium (HWE) for outlier loci of *Pyura chilensis* in the Southeast Pacific coast. In bold, significant deviation of HWE after correction with a False Discovery Rate.

**Table 3:** Population-pairwise F_*ST*_ values for neutral and outlier SNP loci in *Pyura chilensis*.

	PA	CP	LH	TH	LM	AC
PA		0.003	**0.012**	**0.038**	**0.414**	**0.045**
CP	**0.027**		**0.015**	**0.038**	**0.417**	**0.047**
LH	**0.183**	**0.093**		**0.045**	**0.413**	**0.049**
TH	**0.524**	**0.548**	**0.643**		**0.399**	**0.011**
LM	**0.587**	**0.609**	**0.692**	**0.457**		**0.393**
AC	**0.564**	**0.585**	**0.672**	**0.142**	**0.376**	

Genetic differentiation measures of pairwise comparisons calculated using 2251 neutral SNP loci (above the diagonal) and 81 outlier SNP loci (below the diagonal) in *Pyura chilensis*. Significant values after 10,000 permutations (*P* < 0.05) are in bold.

**Table 4:** Analysis of Molecular Variance (AMOVA).

	Neutral Loci		Outlier Loci	
Source of variation	N/S	LM	N/S	LM
Among groups	2.91	**39.51**	**48.73**	8.74
Among populations within groups	**18.4**	**1.84**	**12.51**	**45.47**
Within populations	**78.69**	**58.65**	**38.77**	**45.79**

Percentage of variation explained by different hierarchical levels for neutral and outlier loci considering two groupings in *Pyura chilensis*. One separating North and South of 30°S (N/S) and the other one separating Los Molinos (LM) from the rest of the populations. Significant values in bold.
