# Supplementary material for: Lineage divergence, local adaptation across a biogeographic break, and artificial transport, shape the genetic structure in the ascidian Pyura chilensis
Source: Sci Rep. 2017 Mar 16;7:44559. doi: 10.1038/srep44559 (PMC5353582; doi:10.1038/srep44559)
Supplement: Supplementary Information [file srep44559-s1.docx]

**Supplementary Information**

**Lineage divergence, local adaptation across a biogeographic break, and artificial transport, shape the genetic structure in the ascidian *Pyura chilensis***

Nicolás I. Segovia^1,2,3^, Cristian Gallardo-Escárate^2^, Elie Poulin^3^ & *Pilar A. Haye^1,2^

# ^1^Departamento de Biología Marina, Facultad de Ciencias del Mar, Universidad Católica del Norte, 1781421, Coquimbo, Chile.

^2^Interdisciplinary Center for Aquaculture Research (INCAR), Departamento de Oceanografía, Universidad de Concepción, 4070386, Concepción, Chile.

^3^Instituto de Ecología y Biodiversidad, Departamento de Ciencias Ecológicas, Facultad de Ciencias, Universidad de Chile, 7800003, Santiago, Chile.

***Corresponding author:** Pilar A. Haye, E-mail: phaye@ucn.cl


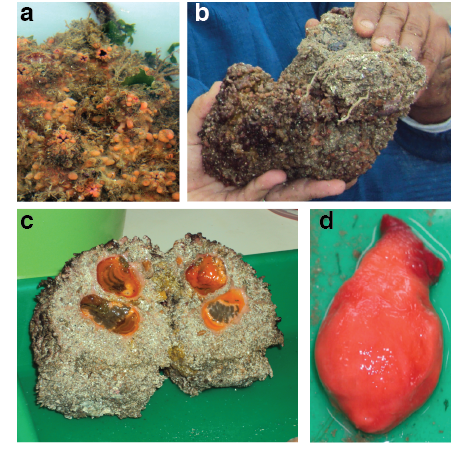


**Figure S1.** ***Pyura chilensis.***

Clump of individuals of *P. chilensis* (a-b), and opened clump with two individuals (c), and an individual after extracting it from the tunic (d), showing its red mantle. Photographs were taken by PAH during samplings.

**Figure S2.** **SNP loci** **F*_ST_* outlier analysis for *Pyura chilensis.***

Results of Bayescan analysis to identify SNPs with outlier F*_ST_* values for 2332 SNPs. The X-axis is the log of the posterior probabilities (q-value) and the dashed line represents the threshold for posterior probabilities using a False Discovery Rate (FDR) of 0.05. Values of q < 0.05 are considered as neutral markers, and *q* > 0.05 as outlier markers.

**
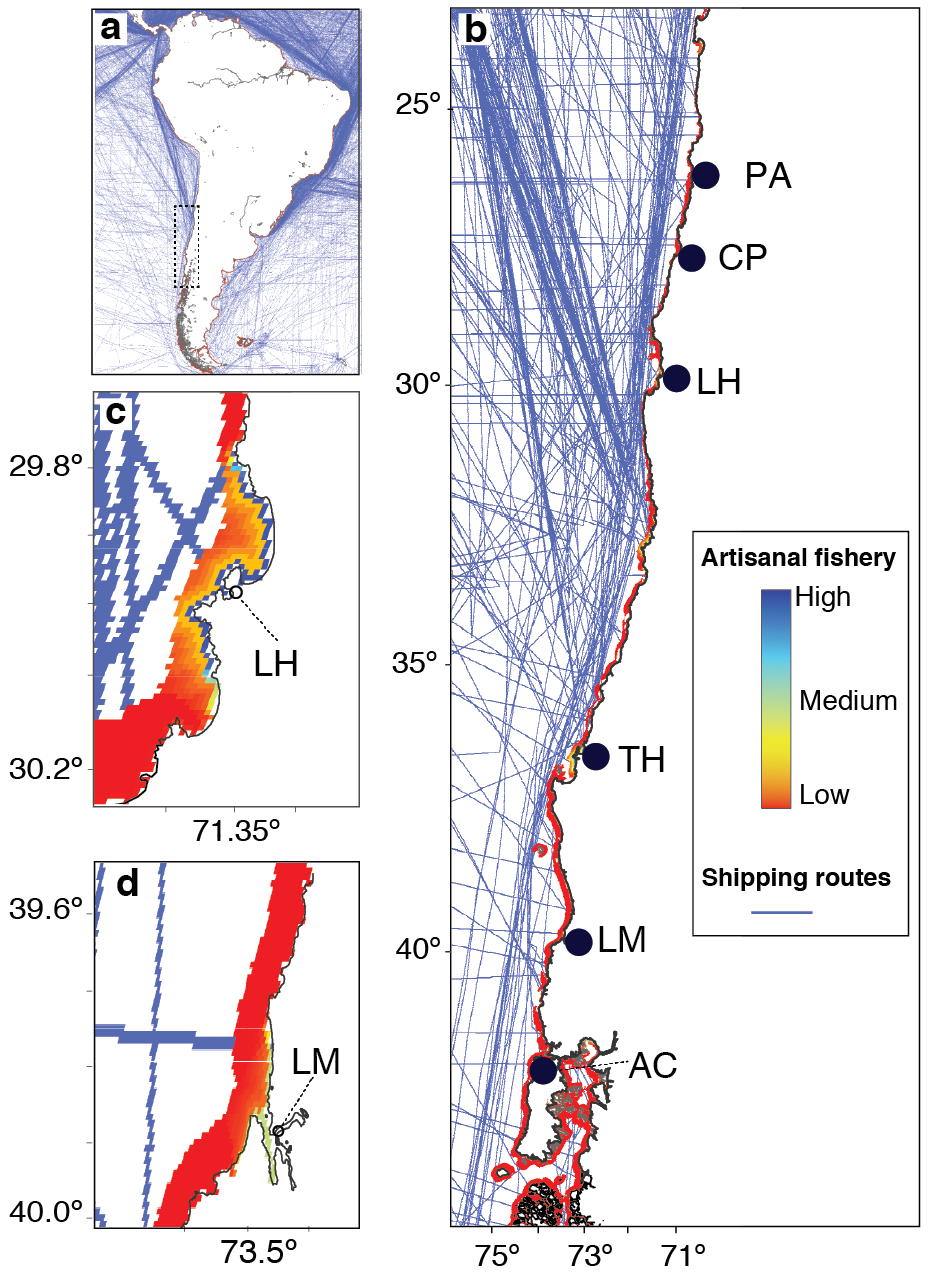
**

**Figure S3. Shipping lines and artisanal fishing routes for the Study Area.**

Maps for marine commercial activity (blue lines) and artisanal fishery modeling (red to blue pixels) were obtained and modified from the Global Map of Cumulative Human Impact project data, available in <https://www.nceas.ucsb.edu/globalmarine/data> (Halpern *et al.* 2015) and plotted in ArcMap 10.4 (ESRI 2011) with the base maps of the coastline of GEODAS, NG, NOAA (<https://www.ngdc.noaa.gov/mgg/shorelines/>), using Coastline Extractor Software to filter the study area (<https://www.ngdc.noaa.gov/mgg/geodas/geodas.html)>. Cumulative influence of commercial and artisanal fishing routes in: (a) South America (data from 2008-2013), (b) detail of the study area, (c) close up of La Herradura (LH) site, and (d) close up of the Los Molinos (LM) site. Scale 1 km^2^ per pixel.

**References**:

- ESRI 2011. ArcGIS Desktop: Release 10. Redlands, CA: Environmental Systems Research Institute.
- Halpern, B. S. et al. Spatial and temporal changes in cumulative human impacts on the world’s ocean. Nat. Commun. 6:7615 doi: 10.1038/ncomms8615 (2015).

**Table S1: Mitochondrial haplogroup assignment**

COI haplogroup assignment of each individual of *Pyura chilensis* analyzed with GBS data according to the haplogroups detected by Haye & Muñoz-Herrera^30^.

| Site | Haplogroup 1 | Haplogroup 2 | Haplogroup 3 |
| --- | --- | --- | --- |
| PA | 15 | 0 | 0 |
| CP | 14 | 1 | 0 |
| LH | 8 | 7 | 0 |
| TH | 6 | 4 | 0 |
| LM | 0 | 3 | 12 |
| AC | 11 | 2 | 2 |
